# Data of thermal imprints of late Permian Emeishan basalt effusion: Evidence from zircon fission-track thermochronology

**DOI:** 10.1016/j.dib.2019.104700

**Published:** 2019-10-22

**Authors:** Di Hu, Yuntao Tian, Jie Hu, Song Rao, Yibo Wang, Chao Zhang, Shengbiao Hu

**Affiliations:** a State Key Laboratory of Lithospheric Evolution, Institute of Geology and Geophysics, Chinese Academy of Sciences, Beijing, 100029, China; b University of Chinese Academy of Sciences, Beijing, 100049, China; c Innovation Academy for Earth Science, Chinese Academy of Sciences, Beijing, 100029, China; d School of Earth Sciences and Engineering, Sun Yat-sen University, Guangzhou, 510275, China; e Key Laboratory of Exploration Technologies for Oil and Gas Resources, Ministry of Education, Yangtze University, Wuhan, 430100, China

**Keywords:** Detrital zircon, Fission-track dating, Emeishan large igneous province, Thermal imprints, Yangtze craton

## Abstract

We present 271 detrital single-grain zircon fission track (ZFT) ages obtained for eight sandstones, which were sampled from the southwestern Yangtze Craton, southern China. Accessory minerals were concentrated using standard crushing, sieving, electro-magnetic and heavy liquid mineral separation techniques. Zircon grains were mounted on FEP Teflon and polished to expose their internal surfaces to 4π geometry. Two to three mounts of each sample were etched in KON:NaOH eutectic melt at ∼228 °C for 12–60 hours to reveal spontaneous fission tracks. Etched mounts were covered with a uranium-free muscovite external detector for the irradiation with thermal neutrons. CN2 standard uranium glasses were embedded with the age standards (Fish Canyon Tuff zircons). After irradiation, external mica detectors were removed from sample mounts and then etched in 48% HF at room temperature for 30 min to reveal induced tracks. Fission track counting was performed using a Zeiss Axiotron microscope at a total magnification of 1250 × . Zircon fission-track ages were calculated using the ζ-calibration technique. The central ages (with 1σ error) vary from 144.7 ± 4.9 Ma to 256.7 ± 9.6 Ma, with variable P(χ2) values of 0%–75%. ZFT ages of the five Cambrian to Ordovician samples are younger than their depositional ages, and thus were fully reset by post-depositional heating. ZFT ages of three Jurassic samples are partially reset, as they overlap with or slightly younger than the corresponding depositional ages.

**Table:** Specifications Table

Subject	Geology
Specific subject area	Thermochronology, which is a branch of geochronology to determine the age of rocks through radioactive isotopes.
Type of data	1 Table 1 Excel file
How data were acquired	Zircon fission-track ages were obtained using external detector method
Data format	Raw Analyzed Filtered
Parameters for data collection	Zircon mounts were etched for 12–60 h at 228 °C in KOH: NaOH eutectic melt. External mica detectors were etched in 48% HF at room temperature for 30 min to reveal induced tracks. Fission track counting was performed using a Zeiss Axiotron microscope at a total magnification of 1250 × .
Description of data collection	Fission track counting was manually performed under laboratory conditions
Data source location	City: Sichuan Country: China Latitude and longitude for collected samples: 28°08ʹ00ʺ(N) to 28°23ʹ39ʺ (N); 103°06ʹ51ʺ(E) to 103°51ʹ03ʺ (E)
Data accessibility	With the article

**Table:** 

**Value of the Data** • The dataset represents thermal imprints of late Permian Emeishan basalt effusion beneath the Yangtze Craton, China. • These ZFT dating also contribute to the thermal evolution of Emeishan mantle plume, which will draw interest from a broad range of researchers in the disciplines of LIP and thermochronology. • The dataset are valuable to regional thermal evolution, which can be further processed by researchers with other geochronological data.

## 1 Data

ZFT dating of eight sandstone samples are compiled in Appendixes A. The dataset contains raw 271 detrital single-grain data through external detector method, as shown in Appendixes B.

## 2 Experimental design, materials, and methods

Eight lithic and quartz sandstone samples were collected. Mineral separation followed the standard density and magnetic procedures after crushing and sieving. All the randomly zircons were mounted in FEP Teflon and polished to expose their internal surfaces to 4π geometry. The detailed method of ZFT analysis followed that of Ref. [1]. Analyses were performed at China University of Geosciences, Beijing.

Two or three mounts of every sample were etched for 12–60 h at 228 °C in a KOH: NaOH eutectic mixture to reveal fossil fission-tracks [2]. Zircon mounts were then covered with a uranium-free muscovite external detector for irradiation in a well-thermalized neutron flux in the 492 Swim Reactor at Beijing. CN2 standard uranium glasses were embedded with the age standards (Fish Canyon Tuff zircons). The external detectors were then etched in 48% HF at room temperature for 30 min to reveal induced tracks. Ages were calculated using the ζ-calibration method [3].

Track counting was performed under a Zeiss Axiotron microscope at a magnification of 1250x. For each sample, we analyzed more than 30 grains. The RadialPlotter software was used to decompose dispersed age data to obtain statistically significant age populations [4].
