# Supplementary material for: Data of thermal imprints of late Permian Emeishan basalt effusion: Evidence from zircon fission-track thermochronology
Source: Data Brief. 2019 Oct 22;27:104700. doi: 10.1016/j.dib.2019.104700 (PMC6838426; doi:10.1016/j.dib.2019.104700)
Supplement: Multimedia component 1 [file mmc1.docx]

Table 1. ZFT dating results for the southwestern Yangtze Craton

| Sample No. | Latitude (N) | longitude (E) | Lithology | Strata | Grains | Ns | Ni | Nd | ζ ±1σ | ρ_d_ | P(χ2) | Central age  (Ma±1σ) | Pooled age  (Ma±1σ) | Dispersion  (%) |
| --- | --- | --- | --- | --- | --- | --- | --- | --- | --- | --- | --- | --- | --- | --- |
|  |  |  |  |  |  |  |  |  | (a cm^2^) | (10^5^/cm^2^) | （%） |  |  |  |
| ZZY-19 | 28°23'39'' | 103°07'00'' | Fine sandstone | J_2_sn | 35 | 6072 | 1977 | 5958 | 90.9 ± 2.8 | 12.37 | 2.2 | 170.1 ± 5.4 | 170.4 ± 7.2 | 11.0 |
| ZZY-18 | 28°21'56'' | 103°06'51'' | Lithic sandstone | J_2_s | 34 | 7542 | 2847 | 5958 | 90.9 ± 2.8 | 11.98 | 0 | 144.7 ± 4.9 | 142.6 ± 5.7 | 14.0 |
| CP-16 | 28°15'59'' | 103°51'24'' | Lithic sandstone | J_1_z | 33 | 9339 | 3347 | 5958 | 90.9 ± 2.8 | 11.59 | 0 | 145.8 ± 4.6 | 145.3 ± 5.7 | 11.0 |
| ZZY-10 | 28°08'00'' | 103°27'07'' | Quartz sandstone | O_1_h | 35 | 8034 | 2151 | 5958 | 90.9 ± 2.8 | 11.20 | 10.0 | 187.6 ± 5.2 | 187.3 ± 7.7 | 7.9 |
| ZZY-9 | 28°08'20'' | 103°27'15'' | Fine sandstone | Є_2x_ | 34 | 9736 | 1974 | 5958 | 90.9±2.8 | 10.80 | 32.3 | 237.3±6.3 | 237.7±9.4 | 5.5 |
| ZZY-6 | 28°09'30'' | 103°27'39'' | Quartz sandstone | Є_1_l | 35 | 9720 | 2660 | 5958 | 90.9±2.8 | 13.74 | 0.1 | 224.5±6.8 | 224.2±9.0 | 12.0 |
| ZZY-2 | 28°10'28'' | 103°27'14'' | Quartz sandstone | Є_1_c | 35 | 6708 | 1871 | 5958 | 90.9±2.8 | 14.13 | 0.8 | 225.0±7.6 | 226.2±9.6 | 12.0 |
| CP-9 | 28°14'25'' | 103°51'03'' | Fine sandstone | Є_1_q | 30 | 3801 | 874 | 5958 | 90.9±2.8 | 13.25 | 75.2 | 256.7±9.6 | 257.0±12.0 | 0 |

*Notes: Ns = number of spontaneous tracks; Ni = number of induced tracks; Nd = number of tracks in the standard uranium glass; ρd = track density in the standard uranium glass; ζ = ζ calibration factor; P(χ2) = chi-squared probability; grains = number of measured grains; 1σ = standard error.*
